# Myocardial ischemia-reperfusion enhances transcriptional expression of endothelin-1 and vasoconstrictor ET_B_ receptors via the protein kinase MEK-ERK1/2 signaling pathway in rat

**DOI:** 10.1371/journal.pone.0174119

**Published:** 2017-03-21

**Authors:** Gry Freja Skovsted, Lars Schack Kruse, Lukas Adrian Berchtold, Anne-Sofie Grell, Karin Warfvinge, Lars Edvinsson

**Affiliations:** 1 Department of Clinical Experimental Research, Glostrup Research Institute, Rigshospitalet, University of Copenhagen, Glostrup, Denmark; 2 Department of Biomedical Sciences, Cellular and Metabolic Research Section, University of Copenhagen, Copenhagen, Denmark; 3 Department of Medicine, Institute of Clinical Sciences in Lund, Lund University, Lund, Sweden; Indiana University School of Medicine, UNITED STATES

## Abstract

**Background:**

Coronary artery remodelling and vasospasm is a complication of acute myocardial ischemia and reperfusion. The underlying mechanisms are complex, but the vasoconstrictor peptide endothelin-1 is suggested to have an important role. This study aimed to determine whether the expression of endothelin-1 and its receptors are regulated in the myocardium and in coronary arteries after experimental ischemia-reperfusion. Furthermore, we evaluated whether treatment with a specific MEK1/2 inhibitor, U0126, modified the expression and function of these proteins.

**Methods and findings:**

Sprague-Dawley rats were randomly divided into three groups: sham-operated, ischemia-reperfusion with vehicle treatment and ischemia-reperfusion with U0126 treatment. Ischemia was induced by ligating the left anterior descending coronary artery for 30 minutes followed by reperfusion. U0126 was administered before ischemia and repeated 6 hours after start of reperfusion. The contractile properties of isolated coronary arteries to endothelin-1 and sarafotoxin 6c were evaluated using wire-myography. The gene expression of endothelin-1 and endothelin receptors were measured using qPCR. Distribution and localization of proteins (pERK1/2, prepro-endothelin-1, endothelin-1, and endothelin ET_A_ and ET_B_ receptors) were analysed by Western blot and immunohistochemistry. We found that pERK1/2 was significantly augmented in the ischemic area 3 hours after ischemia-reperfusion; this correlated with increased ET_B_ receptor and ET-1 gene expressions in ischemic myocardium and in coronary arteries. ET_B_ receptor-mediated vasoconstriction was observed to be increased in coronary arteries 24 hours after ischemia-reperfusion. Treatment with U0126 reduced pERK1/2, expression of ET-1 and ET_B_ receptor, and ET_B_ receptor-mediated vasoconstriction.

**Conclusions:**

These findings suggest that the MEK-ERK1/2 signaling pathway is important for regulating endothelin-1 and ET_B_ receptors in myocardium and coronary arteries after ischemia-reperfusion in the ischemic region. Inhibition of the MEK-ERK1/2 pathway may provide a novel target for reducing ischemia-reperfusion damage in the heart.

## Introduction

Acute myocardial infarction (AMI) is the most common cause of death, with a mortality of more than 6 million people each year worldwide [1]. Treatment strategies aim to restore blood flow using thrombolytic therapy or direct angioplasty with stenting of the affected arteries via percutaneous coronary intervention (PCI). Paradoxically, in addition to the direct ischemic injury, restoring the blood flow can cause damage to the tissue further limiting the beneficial effects of myocardial reperfusion. This phenomenon, termed reperfusion injury, is associated with death of cardiomyocytes that were viable immediately before myocardial reperfusion [2]. The pathogenesis of reperfusion injury involves the interplay of multiple mechanisms, including the release of vasoconstrictors, the no-reflow phenomenon, a profound inflammatory response, apoptosis, and necrosis [3–5]. The coronary vascular endothelium is sensitive to ischemia-reperfusion injury, as manifested by decreased endothelium-dependent vasorelaxation in some models [6], but not in the present model which has been described earlier (Skovsted *et al*. 2014). Comparatively less is known on how ischemia-reperfusion (IR) injury affects the smooth muscle cells in the coronary artery *per se* and in the adjacent myocardium.

The endothelin-1 (ET-1) peptide is an important participant in the pathophysiology of coronary artery disease and myocardial infarction [7]. This peptide is one of the most potent endogenous vasoconstrictors known at present, and it is synthesized and released from vascular and endocardial endothelial cells and from myocytes [8–11]. ET-1 contributes to the regulation of both coronary and peripheral vascular tone [12,13] through its activation of the contractile ET_A_ and relaxant ET_B_ receptors. Moreover, ET-1 release increases during myocardial ischemia and reperfusion, further aggravating this condition. Notably, the plasma levels of ET-1 are increased in patients with coronary artery vasospasm, following myocardial infarction, and in congestive heart failure [14–18]. The vasoconstrictor response to ET-1 is primarily mediated by ET_A_ receptors in vascular smooth muscle cells (VSMCs), and the vasodilator effect is mediated by ET_B_ receptors located in the endothelium [19–21]. We have reported previously on the phenotypic change from relaxant to contractile coronary artery ET_B_ receptors expressed in VSMCs and its increase in human coronary arteries after a fatal myocardial infarct [22] and after experimental myocardial IR *in vivo* [23]. Organ culture of coronary arteries has been used as a surrogate method to study mechanisms involved in the phenotypic alterations of vessel wall receptors. The up-regulation of VSMC ET_B_ receptors is mediated through an increase in transcription and/or translation via the mitogen-activated protein kinase/extracellular signal-regulated kinase (MEK-ERK1/2) signaling pathway [24].

The aim of the present study was to investigate whether the MEK-ERK1/2 signaling pathway is activated early after an IR episode. Furthermore, if this pathway is involved in regulating the expression of ET-1 and endothelin receptors in rat coronary arteries and myocardium after IR using an *in vivo* method, and whether an activation could be attenuated by the MEK1/2 inhibitor U0126.

## Methods

### Animals

Male Sprague-Dawley rats (11–14 weeks old, 330–415 g) were obtained from Taconic, Denmark. The rats were provided with standard rat chow and water *ad libitum* and were housed in a reversed 12 h light/12 h dark condition. All experimental procedures were performed in accordance with national laws and guidelines, and were approved by the Danish Animal Experimentation Board (2012/561-162).

### Experimental protocol

A total of 48 rats were used in the study. They were anesthetized with an induction dose (2.5 ml/kg) of a mixture of Hypnorm-Midazolam (1:1:2) in sterile water (containing 0.079 mg/ml fentanyl, 2.50 mg/ml fluanison, Hypnorm®, VetaPharma Ltd, UK, and 1.25 mg/ml, Midazolam “Hameln”, Hameln, Germany). Anesthesia was maintained using supplementary doses of Hypnorm-Midazolam (1.0 ml/kg) every 20 to 40 minutes. During the surgical procedure, rats were artificially ventilated (75 cycles/min, tidal volume 10 ml air/kg) with a rodent ventilator (Ugo Basile Rodent Ventilator, Comerico, Italy), and body temperature was maintained at 37°C.

Ischemia was induced by ligation of the left anterior descending artery (LAD) for 30 minutes followed by reperfusion or non-occlusion (sham) as described previously [23]. Briefly, the chest was opened through a left thoracotomy, and the heart was exposed by making an incision in the pericardium. A suture was passed around the LAD approximately 4–5 mm away from its origin and tightened. Regional myocardial ischemia was confirmed visually and ST segment elevation was seen on the ECG. After 30 min of ischemia, the ligature was released and the tissue was reperfused. The chest was closed and air was sucked out of the thorax. Thereafter, rats were allowed to recover on a heating pad (37°C) and were extubated when they showed normal spontaneous breathing. For post-operative analgesia and to supplement their fluids, the rats were subcutaneously administered 5 mg/kg carprofen (Rimadyl Vet, Pfizer), 0.03 mg/kg buprenorfin (Temgesic, Schering-Plugh, Brussels, Belgium), and 5 ml 0.9% NaCl isotonic solution immediately after surgery.

**Fig 1:**
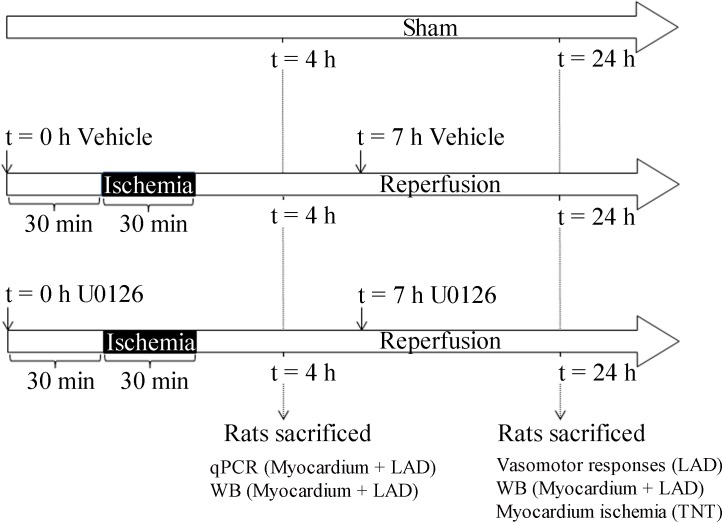
Experimental protocol. A total of 48 rats were allocated into 3 surgery groups: sham, IR+vehicle, and IR+U0126. Rats in the vehicleand U0126 -treatment groups were given intraperitoneal injections with 500 μl DMSO or U0126 (30 mg/kg), respectively, 30 min before ischemia and again subcutaneously 6 hours after reperfusion. Rats were euthanized after either 3 h or 24 h of reperfusion.

The rats were randomly divided into three groups: sham, IR with DMSO (vehicle) and IR with 1,4-diamino-2,3-dicyano-1,4 bis [2-aminophenylthio] butadiene (U0126). Rats were given intraperitoneal injections of U0126 (LC labs, Boston, MA, USA) (30 mg/kg) dissolved in DMSO (Sigma, St Louis, MO, USA) (1.5 ml/kg) or 500 μl DMSO (1.5 ml/kg) 30 min before ischemia and again subcutaneously 6 hours after reperfusion (Fig 1). Rats were euthanized using CO_2_ sedation and decapitation at either 3 h or 24 h of reperfusion. Rats in the 24-h groups were also treated with a per-oral formulation of buprenorphine (Temgesic, Schering-Plough, Brussels, Belgium) 0.4 mg/kg in 5 mg Nutella®.

### Vessel isolation

The heart was immediately after decapitation excised and placed in oxygenated ice-cold physiological saline solution (PSS in mM): 119 NaCl, 4.7 KCl, 2.5 CaCl_2_, 25 NaHCO_3_, 1.17 MgSO_4_, 1.18 KH_2_PO_4_, 5.5 glucose and 0.03 EDTA with 5% CO_2_. LAD and septal coronary arteries (SCA, internal control) were removed by dissecting the tissue around the vessel wall. LAD was divided into two approximately 2-mm segments, one upstream and one downstream of the ligature [23]. SCA segment was dissected out approximately 2–4 mm away from its origin. The endothelium was removed gently by passing a human hair through lumen of the artery segments.

### Functional ex vivo studies

Coronary artery segments from 13 rats (n = 6 in the 24 h IR vehicle group and n = 7 in the 24 h IR U0126 group) were mounted in a wire myograph and stretched to their optimal lumen diameter to obtain optimal conditions for active tension development [23]. ET_B_ receptor-mediated responses were investigated using the sarafotoxin 6c (S6c) peptide, which exhibits high selectivity for ET_B_ receptors compared to ET_A_ receptors [25]. The ET_A_ receptor-mediated response was investigated on the same artery segments after S6c (NeoMPS, Strassbourg, France) by adding ET-1 (NeoMPS, Strasbourg, France) at a concentration range from 10 pM to 0.1 μM. Before adding ET-1, the arteries were pretreated with the specific endothelin ET_B_ receptor antagonist BQ788 (0.9 μM, Sigma, St. Louis, MO, USA) for 30 min. Before each concentration-response curve was determined, the VSMC contractile function was confirmed by challenging the segments two or three times with 125 mM K^+^ (KPSS; similar to PSS except that NaCl was exchanged for KCl on an equimolar basis).

Endothelium function was determined on all artery segments by assessing their relaxation to the acetylcholine receptor agonist carbachol (10 μM) after obtaining steady-state pre-contraction tone with prostaglandin F_2α_ (PGF_2α_) (10 μM). Carbachol-induced relaxation that was <10% of the prostaglandin-induced vasoconstriction was defined as non-functional endothelium.

After the last concentration-response curve was determined, the maximal contractile capacity of each artery was assessed by the addition of a cocktail solution [KPSS plus 10 μM PGF_2α_ and 10 μM 5-hydroxytryptamine (5-HT)].

### Quantitative RT-PCR

Myocardial tissue from the infarcted area of the left ventricle that was downstream of the ligature was isolated from 21 rats (n = 5, 3 h sham group; n = 8, 3 h IR vehicle group; n = 8, 3 h IR U0126 group). The tissue was homogenized using lysing matrices in a FastPrep Cell Disrupter (ThermoSavant) for 3 × 20 s. To prevent heating and degradation, the samples were cooled on ice for a couple of seconds in between each 20-s pulse. RNA extraction was performed according to the NucleoSpin miRNA kit manual from Macherey-Nagel. cDNA was synthesized using an iScript cDNA synthesis kit (BioRad). The real-time PCR amplification was performed with SYBR® Select Master Mix (Life Technologies) using the following primer sets: Ednra (PPR45119A), Ednrb (PPR45420C), and Ppia (PPR06054A) from Qiagen, and ET-1 forward (GAAACAGCTGTCTTGGGAGCAGA) and reverse (CCTTGTCCATCAAGGAGGAGC) from Eurofins. The manufacturer’s guidelines (Qiagen) for the PCR reaction conditions were followed using a ViiA7 PCR machine (Applied Biosystems). The quantity of each gene transcript was quantified using standard curves and normalized to peptidylprolyl isomerase A (Ppia).

### Western blot analysis

Myocardial tissue from the infarcted area of the left ventricle downstream of the ligature was isolated from 26 rats (n = 4, 3 h sham group; n = 4, 3 h IR vehicle group; n = 4, 3 h IR U0126 group; n = 4, 24 h sham group; n = 5, 24 h IR vehicle group; n = 5, 24 h IR U0126 group). After isolation, the tissues were stored at -80°C. For analysis, samples were immersed in 300 μl modified RIPA buffer (50 mM Tris pH 7.5, 150 mM NaCl, 1 mM EDTA, 50 mM beta-glycerolphosphate, 1% NP-40, 0.1% deoxycholate, 0.1% SDS and 0.5% Triton X-100) containing Complete® Protease Inhibitor and PhosStop cocktails (both from Roche, Hvidovre, Denmark) before sonication (2 × 10 1-s pulses, with intermittent cooling on ice, and then 15 s of constant sonication). The resulting lysate was pre-cleared by centrifugation (18,000 × g at 4°C for 15 min). The supernatants were transferred to new tubes, and the total protein concentration was determined using the BioRad DC kit (BioRad Laboratories, Copenhagen, Denmark) and a Tecan M200 spectrophotometer at 750 nm. Samples were adjusted to 20 μg total protein and dissolved in LDS buffer (Expedeon, San Diego, USA) containing 50 mM DTT.

Proteins were separated by gel electrophoresis on 4–20% RunBlue gradient gels (Expedeon, San Diego, USA) at a maximum of 180 V and 150 mA for approximately 1 h 10 min. After electrophoresis, gels were equilibrated briefly in Tris-glycine transfer buffer (2.9 g Trizma-base plus 14.4 g glycine/l, 20% ethanol, and 0.1% SDS diluted in MilliQ water) before transfer to ECL Hybond PVDF membranes (GE Healthcare Life Sciences, Brøndby, Denmark) using wet blotting for 1 h 20 min (maximum settings, 150 V and 350 mA). Membranes were subsequently blocked for 1 h in 2% ECL Prime Blocking Agent (GE Healthcare Life Sciences, Brøndby, Denmark) in TBS-T (TBS plus 0.1% Tween 20) before incubation overnight at 4°C on a rotor with the following primary antibodies diluted in TBS-T containing 0.02% sodium azide: polyclonal rabbit-anti-endothelin ET_B_ receptor antibody (#AER-002, Alomone Laboratories, Israel 1:200); monoclonal mouse-anti-ET-1 antibody (#ab2786, Abcam, 1:1000); polyclonal rabbit-anti-endothelin ET_A_ receptor antibody (#9780, Sigma, 1:1000); rabbit monoclonal anti-total-ERK antibody (#9101, Cell Signaling, 1:2000); or mouse monoclonal anti-pERK antibody (#9107, Cell Signaling, 1:1000). The next day, membranes were washed briefly twice in TBS-T before incubation for 1 h at room temperature with secondary antibodies in TBS-T: either HRP-conjugated donkey-anti-rabbit antibody, 1:40,000 (NA9340V, GE Lifesciences, Brøndby, Denmark) or HRP-conjugated goat-anti-mouse antibody, 1:20,000 (#1858413, Thermo Scientific Pierce, Slangerup, Denmark). Finally, the membranes were washed a minimum of 5 × 5 minutes in large volumes of TBS-T before development using ECL Select Western Blot Detection Kit (GE Lifesciences, Copenhagen, Denmark) for 5 min at room temperature. Image capture was performed using a Fujifilm LAS-4000 imaging unit (Fujifilm, Japan).

To quantify the relative band intensity, the signals were first normalized to GAPDH as an internal loading control and then compared using MultiGauge 3.2 software (Fujifilm, Japan). GAPDH was chosen as a loading control after it was tested for variability in a screen of five possible loading control candidates in an ischemia model (data not shown).

### Co-immunoprecipitation

Freshly isolated myocardium, including the LAD, was homogenized in modified RIPA buffer by sonication (2 × 10 pulses, 30% output power) followed by freezing on dry ice for approximately 1 h and then sonication again. Residual cellular debris was removed by centrifugation (18,000 × g for 15 min at 4°C). Protein concentration measured using the Bio-Rad DC kit; 50 μg of total protein was used for immunoprecipitation (IP), and 25 μg was used as an input control. Each IP reaction was adjusted to a total volume of 300 μl in microfuge tubes by the addition of lysis buffer containing protease and phosphatase inhibitors. Finally, 2 μl of mouse monoclonal anti-ET-1 antibody (#ab2786, abcam, 2.5 μg/μl) was added. Tubes were incubated for approximately 15 h at 4°C on a rotor. The next day, 50 μl of a 50:50 slurry of Protein A/G Sepharose beads resuspended in lysis buffer was added to each IP reaction for 1.5 h. The beads were resuspended in LDS buffer that contained 50 mM DTT. The resulting co-IP reaction and input controls were analyzed as described in the ‘Western blot analysis’ section using rabbit-anti-ET_A_ antibody (#9780, Sigma, 1:500) as the primary antibody and donkey-anti-rabbit antibody (NAV931, GE Lifescience, 1:40.000) as the secondary antibody. Blots were developed using ECL Select followed by LAS-4000 image capture.

### Immunohistochemistry

Immunohistochemical staining was performed on heart tissue that were downstream of the ligature. Tissue from 16 rats (n = 5, 24 h sham group; n = 5 24 h IR U0126 group; and n = 6, 24 h IR vehicle group) were analyzed. The heart tissues were embedded in Tissue-Tek®, frozen on dry ice, and stored at -80°C until further processing. The frozen hearts were sectioned (10 μm) on a cryostat (Leica, Denmark) and mounted on microscope slides (SuperFrost®, Menzel, Germany). The sections were fixed for 20 min using Stefanini’s fixative (2% paraformaldehyde and 0.2% picric acid in phosphate buffer, pH 7.2) and permeabilized in phosphate buffered saline (PBS) containing 0.25% Triton X-100 (T-PBS). To prevent nonspecific staining, sections were blocked with 2% donkey serum and 1% bovine serum albumin (BSA) in T-PBS for 1 h. Samples were incubated at 4°C overnight with primary mouse anti-phosphorylated ERK1/2 antibody (1:200, ab50011, Abcam, Cambridge, UK), sheep anti-endothelin ET_B_ receptor antibody (1:250, alx-210-506a, Alexis Biochemicals, Nottingham, UK) or mouse anti-ET-1 antibody (1:100, ab2786, abcam) diluted in T-PBS. The following day, the slides were rinsed three times in T-PBS and incubated with secondary donkey anti-sheep Texas Red antibody (1:200, Jackson ImmunoResearch, UK) or secondary donkey anti-mouse FITC (1:100, Jackson Immunoresearch Laboratories) for 1 h in room temperature followed by three washes in T-PBS before mounting with Vectashield (Vector Laboratories, Burlingame, CA, USA) medium containing 4’, 6-diamino-2-phenylindole (DAPI, nucleus staining). Primary antibody was omitted as a negative control.

### Myocardial infarction

Blood was collected from aorta of the decapitated rats in BD Vacutainer®SST^TM^ serum tubes (BD Plymouth, UK) at 24h after LAD ligation or sham operation (n = 6 in each group). The blood samples were centrifuged and the serum was immediately stored at -80°C. Serum troponin T was quantified with electrochemiluminescence immunoassay (Elecsys Troponin T STAT 4. gen) at Cobas® 6000 analyzer series (Roche®, Indianapolis, US).

### Statistical analysis

All concentration-response curves were analyzed by non-linear regression analysis using GraphPad Prism 5.03 (GraphPad Corp, SanDiego, CA, USA). Each regression line was fitted to a sigmoid equation: Y = bottom + (top-bottom)/(1+10^((LogEC_50_-X)*Hill Slope)). In this equation, X is the log of the agonist concentration, Y is the contractile response developed by the agonist and normalized to the cocktail response, ‘bottom’ is the initial contractile response (basal tone), and ‘top’ is the top plateau of the concentration response curve induced by the agonist. ‘Hill Slope’ describes the steepness of the curve. E_max_ is the difference between the top and bottom normalized to the cocktail response, and sensitivity to the agonist is expressed as the negative logarithm of the molar concentration of the agonist that elicited 50% of the maximum contraction (pEC_50_). The results are reported as the mean ± standard error of the mean (SEM); n denotes the number of rats. Statistical analysis of qPCR, Western blot and serum Troponin T were performed by one-way ANOVA followed by Bonferroni’s multiple comparison test. Coronary contraction in response to S6c or ET-1 were compared in the different vascular segments by two-way repeated-measures ANOVA followed by Bonferroni post-test (when applicable). Differences were considered statistically significant for *P* < 0.05.

## Results

### IR treatment groups

Rats underwent IR according to the protocol shown in Fig 1. The three surgery groups were: sham operated group, IR vehicle group, and IR with U0126 (i.p. 30 mg/kg) group. Rats were treated with vehicle only or U0126 (s.c. 30 mg/kg) 30 min before occlusion and 6 h after reperfusion and euthanized either 3 h or 24 h after start of reperfusion. During the surgical procedure, rats were monitored with ECG electrodes. Immediately after the LAD occlusion, the ECG changed to a typical pattern with ST-elevation that persisted during the ischemic period. After reperfusion, the ST-elevation gradually normalized within 3 h (data not shown).

### Protein kinase activation / phosphorylated ERK1/2 analysis

**Fig 2:**
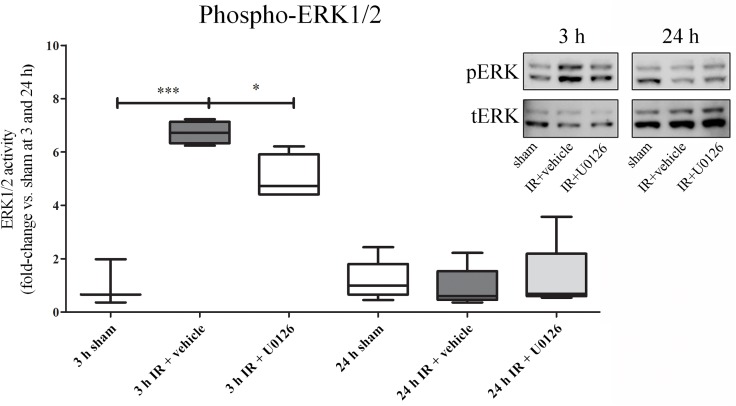
Western blot analysis of heart homogenates that contained myocardium and the left anterior descending artery (LAD). **A.** There was a significant increase in ERK1/2 phosphorylation as normalized to the total ERK1/2 at 3 h of reperfusion, and this was significantly attenuated by treatment with U0126. After 24 h, phospho-ERK levels had returned to baseline and no changes were seen between the groups. Western blot results are shown as dot plots, where each dot represent an individual rat (n = 4–5). Bars and whiskers indicate mean values ± SEM. *P < 0.05, ***P < 0.001, one-way ANOVA with Bonferroni’s multiple comparison test.

To determine whether IR induced activation of the MEK-ERK1/2 signaling pathway, we performed phospho-specific Western blot analysis of homogenates of myocardial tissue that contained a segment of LAD downstream of the occlusion. Quantitative analysis of phosphorylated (p) ERK1/2 revealed a significant increase after ischemia at 3 h of reperfusion (p<0.001), which was significantly attenuated by MEK1/2 inhibitor U0126 (p<0.01). At 24 h of reperfusion, no difference was observed for pERK1/2 between sham, vehicle or U0126 groups (Fig 2A).

### Endothelin expression levels

**Fig 3:**
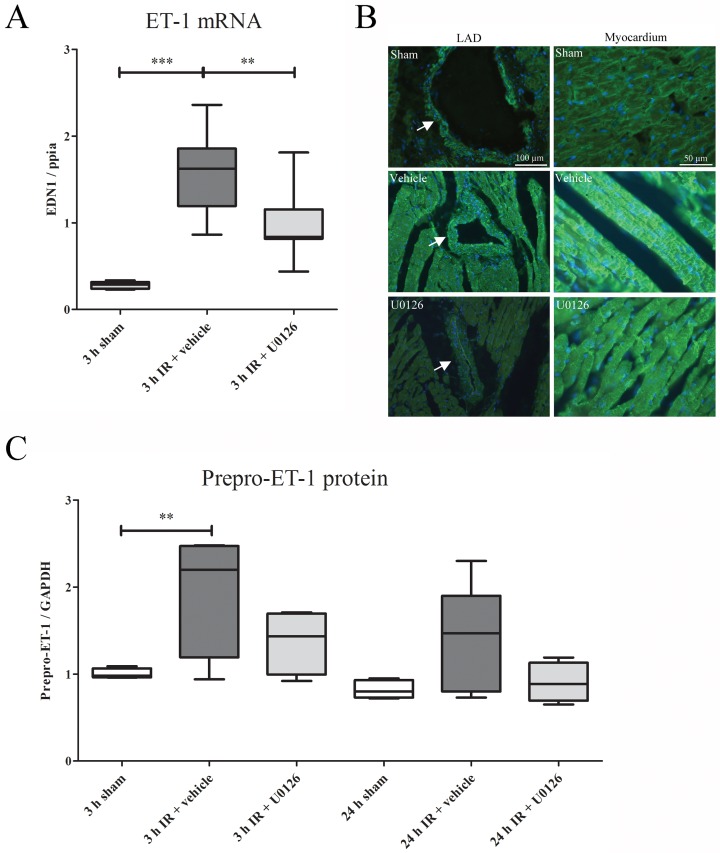
**(A)** The mRNA expression levels of ET-1 were sigificantly upregulated at 3 h of IR. U0126 treatment decreased ET-1 mRNA levels significantly compared to vehicle treatment. Results are shown as whisker plots, with whiskers indicating SEM (n = 5–8), ** *P* ≤ 0.01, ***P ≤ 0.001, one-way ANOVA with Bonferroni’s multiple comparison test. **B.** Protein immunohistochemistry of LAD (left column, arrows) and myocardium (right column) revealed an increase of ET-1 expression in the vehicle treatment after 24 h of IR. This elavated expression was decreased after treatment with U0126 in both LAD and in the myocardium. **(C)** Western blot analysis revealed a significant increase in prepro-ET-1 protein levels (30 kDa) at 3 h of IR, and the same trend was observed at 24 h of IR. The results are shown as Whisker box plots, with bars and whiskers indicating mean values ± SEM (n = 4–5). ** *P* ≤ 0.01, one-way ANOVA with Bonferroni’s multiple comparison test.

There was a strong increase in ET-1 expression in the LAD and myocardium segments distal to ligation in rats subjected to IR and vehicle treatment (Fig 3). The ET-1 mRNA (Fig 3A) level was measured by qPCR and found to be significantly elevated at 3 h of reperfusion (p<0.001). Treatment with U0126 significantly reduced the expression of ET-1 mRNA (p<0.005). In the vehicle treated animals, immunohistochemistry revealed an increase of ET-1 expression in VSMCs and in the myocardiumafter 3 h of reperfusion compared to sham. This elevated expression was decreased in animals treated with U0126 (Fig 3B).

Western blot analysis using an antibody against an epitope containing amino acids 8–16 of ET-1 showed the presence of the prepro-ET-1 peptide (Fig 3C). In contrast to the mature form of ET-1, which is too small to be detected, prepro-ET-1-like reactivity appeared as a 30-kDa band. Quantification of the Western blots revealed a significantly increased prepro-ET-1 level at 3 h of reperfusion as well as a non-significant (p = 0.06) increase at 24 h of reperfusion. The pattern of changes were similar at the two time-points. We found no significant differences in prepro-ET-1 peptide levels in rats treated with U0126 compared to sham-treated animals at 3 and 24 h of reperfusion, thus it had normalized to the sham level (Fig 3C).

### Coronary artery vasomotor responses

In order to measure the vasoconstrictor responses independent of endothelial function, the endothelial layer was removed gently after the coronary artery segments were dissected free of adjacent myocardial tissue. The absence of endothelial function was confirmed by a lack of carbachol-induced relaxation in pre-constricted artery segments. The average contractile capacity responses measured after adding the pharmacological cocktail [KPSS+ 5-HT (10 μM) plus PGF_2α_ (10 μM)] were not significantly different in the SCA compared with those in the LAD, and there was no difference in the contractile capacity of coronary artery segments from rats treated with vehicle versus U0126 (data not shown).

### Endothelin receptor pharmacology

**Fig 4:**
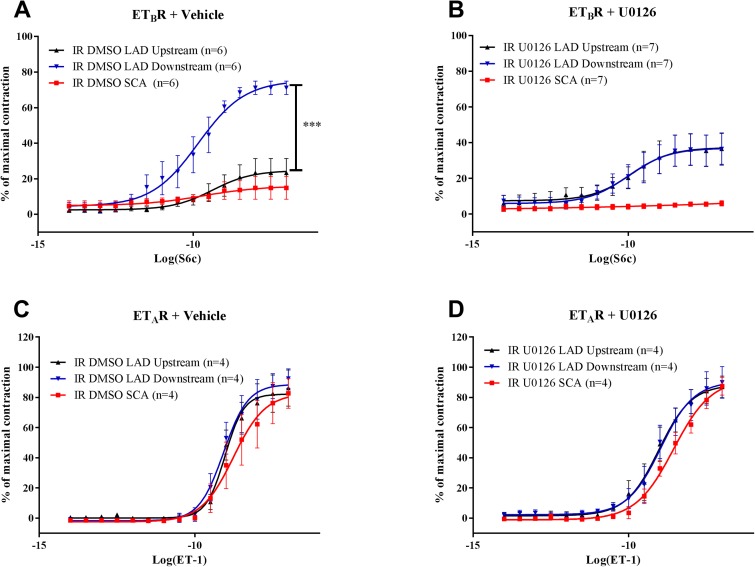
Concentration-response curves show the effect of increasing concentrations of Sarafotoxin 6c (S6c) in the left anterior descending artery (LAD) upstream of the ligature, LAD downstream of the ligature, and non-ischemic septal coronary artery (SCA) after treatment with **(A)** IR and vehicle or **(B)** U0126. The results are shown as mean values ± SEM (n = 6–7), ***P < 0.001; two-way ANOVA with repeated measurements; LAD downstream vs. upstream. Concentration-response curves show the effect of increasing concentrations of endothelin-1 (ET-1) after ET_B_ receptor desensitization and BQ788 treatment in the LAD upstream of the ligature, LAD downstream of the ligature, and non-ischemic SCA after treatment with **(C)** the vehicle or **(D)** U0126. The results are shown as mean values ± SEM (n = 4–7), ***P < 0.001; two-way ANOVA with repeated measurements; LAD downstream vs. upstream.

The vasoconstrictor responses to S6c and ET-1 (the latter after de-sensitization of the ET_B_ receptors; Skovsted *et al*. 2014, Adner *et al*. 1996) were studied in endothelium-denuded coronary artery segments isolated 24 h after IR (Fig 4). S6c induced negligible vasoconstriction in non-ischemic coronary artery segments (LAD segments upstream of the occlusion and SCA segments) (Skovsted *et al*. 2014). In contrast, strong S6c-induced vasoconstriction was seen in distal coronary artery segments isolated from the ischemic zone downstream of the occlusion in vehicle-treated animals (Fig 4A). U0126 treatment abolished IR-induced functional increases in ET_B_ receptor-mediated vasoconstrictor responses in downstream artery segments (Fig 4B).

The ET_A_ receptor-mediated responses to ET-1 were strong and concentration-dependent. In general, the LAD segments from both vehicle- and U0126-treated rats were slightly more sensitive to ET-1 than those of the SCA (Fig 4C and 4D). However, there were no changes in vasoconstrictor responses in LAD segments from the post-ischemic zone compared to the non-ischemic zone, and U0126 did not modify ET_A_-receptor-mediated contractions (Fig 4C and 4D).

### Endothelin receptor expression

#### ET_A_ receptor

**Fig 5:**
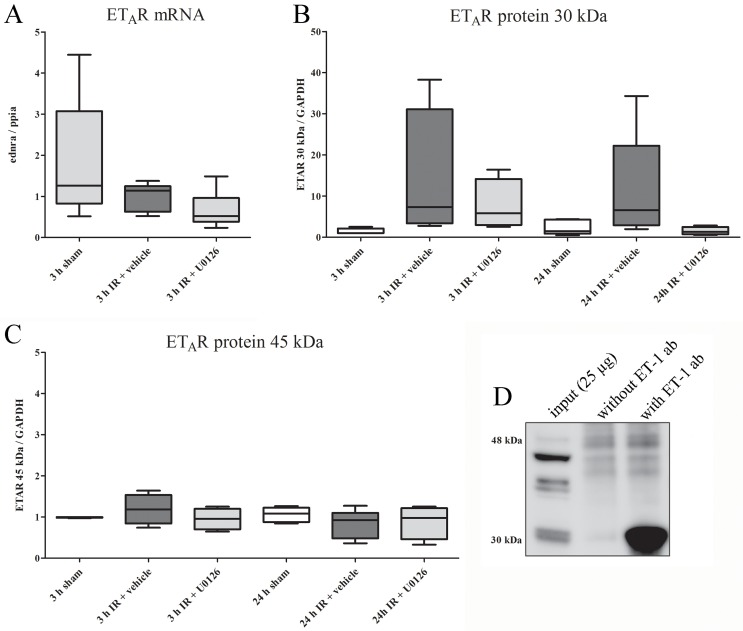
**(A)** mRNA expression levels of ET_A_R in homogenates of the myocardium and left anterior descending artery (LAD) from sham, IR, or IR and U0126 treated rats at 3 h of reperfusion. No significant changes in ET_A_R mRNA expression were found between the sham, IR, and IR+U0126 groups (one-way ANOVA post-Bonferroni’s multiple comparison test). Results are expressed as whisker box plots (n = 5–8). **(B)** Two ET_A_R-like immunoreactive bands were observed. The upper (higher molecular weight) band, corresponding to full-length ET_A_R (45 kDa), did not show any changes in overall expression level regardless of treatment and reperfusion duration (one-way ANOVA post-Bonferroni’s multiple comparison test). Results are shown as Whisker boxplots, (n = 4–5). **(C)** The lower molecular-weight band (30 kDa), generally showed strong up-regulation at 3 h of reperfusion, and this was partly retained at 24 h. U0126 tended to reduce this increase, although this was not significant. Results are shown as Whiske box plots (n = 4–5). **(D)** A co-immunoprecipitation experiment used 25 μg total protein (3 h IR + vehicle) as input and 5 μg mouse mAb anti-ET-1 (Abcam, ab2786) as the immunoprecipitating antibody. Western blot analysis was performed to detect the ET_A_R using Sigma pAb rabbit-anti-ET_A_R #9780 1:500. Specific ET_A_R-like immunoreactivity was observed for the low molecular weight band (30 kDa).

We found no significant changes in ET_A_ receptor mRNA expression at 3 h of reperfusion in any of the experimental groups (Fig 5A). In addition, there were no changes in the levels of the full-length (45 kDa) ET_A_ receptor protein as analyzed by Western blot at 3 or 24 h (Fig 5B). However, a low molecular-weight band at 30 kDa generally showed up-regulation at 3 h of reperfusion, and up-regulation was partly retained after 24 h of reperfusion (Fig 5C). Interestingly, this low molecular weight ET_A_ receptor-like immunoreactive protein bound to ET-1, since this band was observed exclusively as a co-immunoprecipitated protein using an antibody targeting ET-1; it was absent when the antibody was omitted from the co-immunoprecipitation reaction (Fig 5D, lanes 3 and 2, respectively).

#### ET_B_ receptor

**Fig 6:**
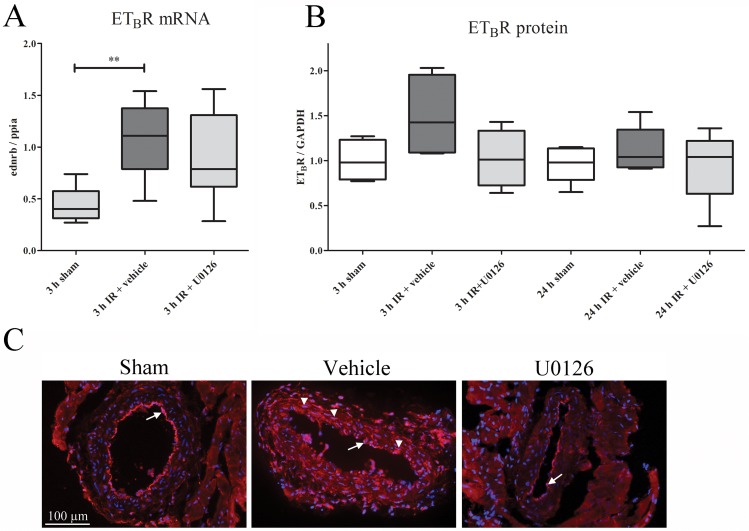
**(A)** The mRNA expression levels of ET_B_R in the myocardium and left anterior descending artery (LAD) homogenates in sham, ischemia-reperfusion (IR), and IR+U0126 treated rats at 3 h of IR resulted in up-regulated ET_B_R mRNA compared to sham. After U0126 treatment, there was no significant upregulation as compared to sham (n = 5–8) (one-way ANOVA with Bonferroni’s multiple comparison test). **(B)** Western blot analysis revealed an ET_B_ receptor band at approximately 50 kDa. Quantification of ET_B_ receptor immunoreactivity (normalized to GAPDH) revealed increased ET_B_ receptor protein levels (*P < 0*.*05*) at 3 h in the IR group compared to the sham group. This was normalized in the U0126 treated group. No significant differences were found between the remaining groups (one-way ANOVA). **(C)** Immunohistochemistry of the 24 h IR group showed the localization of ET_B_Rs in the endothelium (arrow) of sham operated rats. In the IR+vehicle group, immunoreactivity in the VSMCs (arrow heads) in addition to the endothelium (arrow) was found. In contrast, ET_B_R staining was absent in the VSMCs of the IR+U0126-treated rats.

In post-ischemic LAD and adjacent myocardial tissue, ET_B_ receptor mRNA had increased significantly at 3 h after reperfusion (Fig 6A). ET_B_ receptor protein levels increased significantly at 3 h of reperfusion, as compared to sham, however afterU0126 treatment ET_B_ receptor protein level was no significantly increased compared to sham. The ET_B_ receptor expression showed a similar pattern after 24 h of reperfusion compared to tissue from sham-operated rats (Fig 6B). With immunohistochemistry we found increased ET_B_ receptor-like immunoreactivity that localized exclusively to VSMC of LAD coronary arteries after 24 h of reperfusion (Fig 6C). Treatment with U0126 attenuated the significant changes in ET_B_ receptor mRNA expression (Fig 6A). The ET_B_ receptor-like immunoreactivity in VSMCs found in the vehicle group was abolished in the U0126 group (Fig 6C). As expected there was a weak ET_B_ receptor expression in the endothelium but this was unaltered by IR (Fig 6C). The adjacent myocardium did not show any changes in the expression of ET_B_ receptors.

### Myocardial infarction

**Fig 7:**
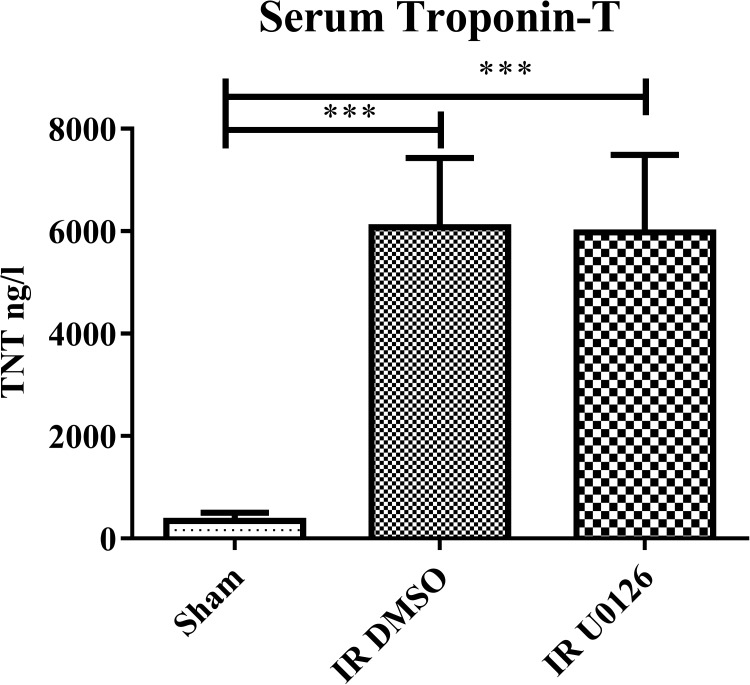
Serum Troponin T measurements in sham operated, IR+vehicle and IR+U0126 (n = 6 in each group). ***P < 0.001, by one-way ANOVA followed by Bonferroni’s multiple comparison test.

Myocardial ischemic damage was measured as serum levels of cardiac troponin T.We observed that LAD ligation resulted in a marked significant increase in serum Troponin T levels, but U0126 showed no a reduction in the serum troponin T level (Fig 7).

## Discussion

The present study is the first to demonstrate *in vivo* that IR up-regulates the expression of both ET_B_ receptors and cellular ET-1 in the distal post-ischemic part of the occluded artery wall of the LAD and in adjacent myocardium, and that this process is regulated by the MEK-ERK1/2 pathway. The MEK-inhibitor (U0126) attenuated ET-1 expression and ET_B_ receptor expression in post-ischemic hearts. Analysis of serum troponin levels 24 h after IR revealed a strong increase while at this early stage after the ischema U0126 did not reduce this significantly. This response is not unexpected because in methods of global cerebral ischemia (Johansson et al., 2014) and in experimental subarachnoid hemorrhage (Edvinsson and Povlsen, 2011) the MEK1/2 inhibition had a pronounced effect on late cerebral ischemia and on neurology outcome and survival several days after the injury. We hypothesise that the coronary ischemia approach may reveal a similar long-term effect on heart function and this deserves future analysis.

The ET system in the circulation has a clear role in human pathophysiology with (i) expression in myocardium and coronary vasculature and (ii) association with e.g. hypertension, heart failure, kidney disease and diabetes (Davenport *et al*. 2016). Circulating levels of the strong and potent ET-1 is increased in myocardial infarction [14,26]. Further, plasma ET-1 concentrations are strongly linked to outcome after myocardial infarction and can provide information that is associated with a poor prognosis [27] as well as to congestive heart failure [28]. Enhanced ET-1 levels during heart ischemia may result in increased coronary artery tone and that may further impair coronary blood flow. Synthesis of the biologically active 21-amino acid ET-1 peptide is a multi-step process where the transcriptional regulation of the ET-1 gene (*edn1*) is thought to be the major mechanism controlling ET-1 bioavailability [29]. Both hypoxia and increased shear stress, important factors in ischemia and reperfusion, are known to increase ET-1 expression and release [30–32]. Many signaling pathways are involved in *edn1* gene regulation, including pathways that include protein kinase C and the MAPKs JNK and ERK1/2 [33,34]. Here we report that ET-1 itself was up-regulated in the myocardium and in the walls of coronary arteries after IR. In addition, treatment with U0126 reversed the increase in ET-1, which is novel and strongly suggests that signaling via the MEK-ERK1/2 pathway is involved in transcriptional up-regulation of ET-1 in the myocardium after IR.

Freshly isolated and healthy rat coronary arteries show negligible ET_B_ receptor-mediated contractions [35,36] but can induce relaxation via endothelin ET_B_ receptors. In ischemia there is a phenotypic shift and vasoconstrictor endothelin ET_B_ receptors are expressed in tissues affected by cardiovascular diseases [22]. In the present study, non-ischemic coronary arteries displayed negligible vasoconstrictor responses to S6c, while post-ischemic arteries showed strong concentration-dependent constriction. The enhanced ET_B_ receptor-mediated vasoconstrictor responses in the downstream LAD were evident at 24 h [23]. The enhanced ET_B_ receptor mRNA expression was verified by qPCR already at 3 h of reperfusion, and the ET_B_ receptor protein up-regulation was seen to occur in VSMC by immunohistochemistry at 24 h. Up-regulation of functional ET_B_ receptors may take several hours, as shown in an *ex vivo* organ culture method, and ET_B_ receptor up-regulation involves activation of the MEK-ERK1/2 signaling pathway [24]. The present study demonstred this *in vivo* using the specific MEK1/2 inhibitor U0126. After treatment with U0126, the up-regulation of vasoconstrictor ET_B_ receptors was abolished at 24 hours, which is in concert with the immunohistochemistry results.

The endogenous ligand to endothelin receptors ET-1 has equally high affinity for ET_A_ and ET_B_ receptors [37]. Since ET_B_ receptors desensitize rapidly upon S6c binding [38], we were able to distinguish between ET_A_ and ET_B_ receptor mediated vasoconstrictor responses by first stimulating ET_B_ receptors with the specific agent S6c and, after desensitization of ET_B_ receptors, blocking the remaining ET_B_ receptors with BQ788 (Adner *et al*. 1996). We then measured ET_A_ receptor-mediated vasoconstriction using ET-1 and found that ET_A_ receptor-mediated responses remained unaffected in post-ischemic coronary arteries at 24 h after IR. Interestingly we observed two main ET_A_R-like immunoreactive bands in our Western blot analysis. The upper band, which corresponds to full-length ET_A_R (426 aa) at approximately 45 kDa, did not show any significant changes in expression level regardless of IR, treatment, or duration of reperfusion. In contrast, the low-molecular weight band, at approximately 30–33 kDa, showed a strong trend towards upregulation during the initial phase after IR, and this persisted after 24 h. The nature of the latter band remains unclear, but a pilot co-IP reaction (Fig 5D) identified this band as the primary co-immunoprecipitated protein when the reaction was performed with a monoclonal antibody against ET-1/prepro-ET-1 followed by blotting to detect the ET_A_ receptor. In contrast, qPCR analysis did not reveal any global changes in ET_A_ receptor mRNA. This analysis utilized a primer set located within exon 2, which contains part of the 5’-UTR, the signal peptide sequence, and the first 120 amino acids of the receptor. As the signal peptide is likely to be crucial for membrane insertion, this exon is probably contained in all putative splice variants of ET_A_. Thus, the primer set should measure the combined pool of ET_A_ mRNA transcripts. In addition, if the up-regulation we observed was mainly due to changes in the rate of translation, no changes in mRNA levels would be expected. A number of ET_A_ receptor splice variants have been reported [39–43], few of which have been confirmed as functional proteins [40]. Most studies report a truncated version in which exons 3 and/or 4 have been spliced out, resulting in proteins with 2 to 5 transmembrane domains. In the absence of exons 3 and 4, this would produce a peptide of the size observed in this study; however, this truncated version, when expressed in CHO cells, did not show any binding affinity for either ET-1 and ET-3 [41]. Given the robust changes we observed in the low-molecular weight band and the reported presence of various splice variants of the ET_A_ receptor, we speculate that this band may be a novel ET_A_ receptor splice variant that retains ET-1 binding capacity but lacks vasoconstrictive effects. Further studies are needed to confirm this.

Occlusion of a coronary artery results in insufficient flow to the contracting myocytes and, if the ischemia is prolonged, it results in myocardial cell death. Rapid PCI can restore epicardial blood flow, but has some drawbacks. Although reperfusion reduces infarct size and enhances survival, it may in itself result in reversible injury, stunning, arrhythmias, and irreversible reperfusion injury. Thus, reperfusion may account for about 50% of the final infarct size [44]. Hence, our main aim with this study was to evaluate if the occlusion and reperfusion of LAD might be associated with alterations in the expression of ET-1 and its receptors, and if inhibition of this upregulation might modify the resulting tissue damage. Increased expression of the ET-1 ligand and its receptors, along with elevated vasoconstriction, may explain the diminished blood flow, at least in part. A more detailed functional analysis of the long-term consequences of the prevention of the vascular phenotypic shift in ET-1 and ET_B_ receptor expression, will be done using functional studies with high field MRI. Understanding the underlying molecular mechanisms involved may provide novel ways to reduce tissue injury after IR. We propose that inhibiting both the elevation of ET-1 and ET_B_ receptor expression in conjunction with IR could be beneficial in terms of improving coronary blood flow and attenuating coronary artery injury.

In summary, we found that treatment with the specific MEK1/2 inhibitor U0126 reduced the early activation of pERK1/2 in the vessel wall after IR at 3 hrs and in addition attenuated the *de novo* expression of both vasoconstrictor ET_B_ receptors and the expression of ET-1 after myocardial IR,. These novel findings suggest that the MEK-ERK signaling pathway is important for regulation of ET-1 and ET_B_ receptor expression and function in the myocardium and coronary arteries after myocardial IR.

## Abbreviations

BQ788: *N*-*cis*-2,6-dimethylpiperidinocarbonyl-L- γ-methylleucyl- D-1-methoxycarboyl-D-norleucine
ERK1/2: Extracellular signal-regulated kinase 1/2
ET_A_ receptor: endothelin receptor type A
ET_B_ receptor: endothelin receptor type B
ET-1: endothelin-1
IR: ischemia-reperfusion
LAD: left anterior descending coronary artery
MAPK: mitogen-activated protein kinase
MEK1/2: mitogen-activated protein kinase kinase 1/2
S6c: sarafotoxin 6c
SCA: Septal coronary artery
U0126: 1,4-diamino-2,3-dicyano-1,4-bis[2-aminophenylthio] butadiene
VSMC: vascular smooth muscle cell
